# Energy Devices for Clipless–Sutureless Laparoscopic Appendectomy: A Systematic Review and Meta-Analysis on Utility and Safety

**DOI:** 10.3390/medicina58111535

**Published:** 2022-10-27

**Authors:** Apoorv Singh, Sachit Anand, Niklas Pakkasjärvi, Ajay Verma, Minu Bajpai

**Affiliations:** 1 Department of Pediatric Surgery, All India Institute of Medical Sciences, New Delhi 110029, India; 2 Department of Pediatric Surgery, Turku University Hospital and University of Turku, 20520 Turku, Finland; 3 Department of Pediatric Surgery, Uppsala Akademiska Sjukhuset, 75185 Uppsala, Sweden

**Keywords:** clipless–sutureless laparoscopic appendectomy, acute appendicitis, postoperative outcomes

## Abstract

*Background and Objectives*: While laparoscopic appendectomy is standardized, techniques for appendiceal stump closure and mesoappendix division remain variable. Novel vessel sealing techniques are increasingly utilized ubiquitously. We sought to systematically summarize all relevant data and to define the current evidence on the safety and utility of energy devices for clipless–sutureless laparoscopic appendectomy in this systematic review and meta-analysis. *Materials and Methods*: This review was conducted following the PRISMA guidelines. PubMed, Embase, Scopus, and Web of Science were systematically searched. Inclusion criteria included studies with laparoscopic appendectomy for appendicitis. The intervention included patients undergoing division of mesoappendix and/or securing of the appendicular base using diathermy (Monopolar or Bipolar or LigaSure Sealing Device) or Harmonic Scalpel (Group A) compared to patients undergoing division of mesoappendix and/or securing of the appendicular base using endoclip or Hem-o-lok or ligature (Group B). The methodological quality of the included studies was assessed using the Downs and Black scale. The outcomes of surgical site infection (SSI) or intra-abdominal collection, postoperative ileus, average operative duration, and length of hospital stay (LHS) were compared. *Results*: Six comparative studies were included; three were retrospective, two were prospective, and one was ambispective. Meta-analysis revealed a shorter operative duration in Group A with respect to appendicular base ligation (MD −12.34, 95% CI −16.57 to −8.11, *p* < 0.00001) and mesoappendix division (MD −8.06, 95% CI −14.03 to −2.09, *p* = 0.008). The pooled risk ratios showed no difference in SSI between groups. Additionally, no difference was observed in LHS. The risk of postoperative ileus was higher in group B regarding mesoappendix division (RR 0.56, 95% CI 0.34 to 0.93, *p* = 0.02), but no difference was found concerning appendicular base ligation. The included studies showed a moderate-to-high risk of bias. *Conclusions*: Clipless–sutureless laparoscopic appendectomy is safe and fast. Postoperative ileus seems less common with energy devices for mesoappendix division. However, the studies included have a moderate-to-high risk of bias. Further studies addressing the individual devices with surgeons of similar levels are needed.

## 1. Introduction

Laparoscopic appendectomy was introduced in 1983 and has since become the operative method of choice for both uncomplicated and complicated appendicitis [1,2]. Laparoscopic appendectomy has proven safe for all ages, even for complicated or perforated appendicitis [3]. However, laparoscopic appendectomy entails a somewhat higher intra-abdominal abscess percentage than traditional open appendectomy [4]. In traditional open appendectomy, the appendiceal stump is transfixed and usually inverted into the cecum, but this has been omitted in laparoscopic appendectomy, and the stump is closed according to the surgeon’s preference. Ligation of mesoappendix in open surgery has also been replaced by electrocautery in laparoscopic appendectomy. It has been proposed that the method of appendiceal stump closure would affect postoperative infections [5]. The current evidence regarding the optimal methods of stump closure and mesoappendix ligation in laparoscopic appendectomy remains controversial. Stump closure can be achieved through either ligature techniques or mechanical devices, and mesoappendix division by simple electrocautery or mechanical devices. It remains unclear whether the higher cost of mechanical devices is nullified by time savings and safer closure. However, thus far, overall complications do not seem to depend on the method of appendiceal stump closure [6].

Novel vessel-sealing devices such as electrothermal bipolar-activated LigaSure and ultrasonic systems such as Harmonic are widely utilized in surgery and are becoming more popular [7,8]. These devices have different modes of action, with LigaSure using a combination of pressure and electrothermal energy to occlude vessels and Harmonic denaturing and coagulating collagen fibers by applying mechanical ultrasonic energy and thereby leading to vessel obstruction. The introduction of these devices has shortened operative times in many fields [9]. In thyroid surgery, Harmonic has proven to be faster without affecting postoperative morbidity. Similarly, in colorectal surgery, LigaSure has resulted in shorter operative times [10,11]. However, thus far, no device has proven to be superior to others overall.

The Harmonic scalpel exposes the surrounding tissues to less energy than other sealing devices and has been shown to be an alternative for appendiceal stump closure [12,13,14]. We initiated this systematic review and meta-analysis to investigate the current evidence for the methods of appendiceal stump closure and mesoappendix division during appendectomy in both children and adults using the currently available sealing devices, including the harmonic scalpel. We believe this is the first published meta-analysis on this subject.

## 2. Materials and Methods

### 2.1. Search Strategy

The current systematic review and meta-analysis were performed in accordance with the Preferred Reporting Items for Systematic Reviews and Meta-Analyses (PRISMA) guidelines without PROSPERO registration [15]. Two authors (SA and NP) performed the initial search of the PubMed database on 24th March 2022 to identify the already published literature and rule out the presence of any existing meta-analysis on the research topic. Subsequently, a systematic literature search was performed by both the authors of the four databases including PubMed, Web of Science, EMBASE, and Scopus. The following keywords were utilized for performing the search: (sutureless OR clipless OR knotless OR harmonic OR sealing OR diathermy OR no suture OR no clip) AND (appendicectomy OR Appendectomy OR appendicular stump). The duplicate records were subsequently discarded from the search results, and the remaining studies were analyzed for eligibility.

### 2.2. Eligibility Criteria

The inclusion criteria applied were: *participants*—studies where laparoscopic appendectomy was performed for cases with appendicitis; *intervention*—patients undergoing division of mesoappendix and/or securing of the appendicular base using diathermy (Monopolar or Bipolar or LigaSure Sealing Device) or Harmonic Scalpel (Group A); *comparison*—patients undergoing division of mesoappendix and/or securing of the appendicular base using endoclip or Hem-o-lok^®^ or ligature (Group B); *outcomes*—the proportion of patients developing surgical site infection (SSI) or intraabdominal collection, the proportion of patients developing postoperative ileus, average operative duration, and average duration of hospital stay were compared among the two patient groups.

Studies that reported at least one of the aforementioned outcomes were included. No specific age criteria were applied, and studies with both children and adults were included. Non-comparative studies, case reports, editorials, letters to the editors, opinion articles, and conference abstracts were excluded. In addition, studies with unavailable full texts were also excluded.

### 2.3. Data Synthesis

Two investigators (AS and SA) executed the data extraction independently in Microsoft Excel (Version 2205) spreadsheets. Any disagreements among them were settled through consensus or discussion with another investigator (NP). Apart from the data on the above-mentioned outcomes, information regarding the name of the author, year of publication, type of study design, number of patients assessed in each study, and the number of patients in each treatment group were extracted.

### 2.4. Quality Assessment

Two investigators (AS and SA) independently assessed the methodological quality of the included studies utilizing the validated Downs and Black scale [16]. This is a twenty-seven-item questionnaire with four domains and a total score ranging from 0 to 32. Based on the total scores assigned, each study was graded to have a risk of bias as high risk (Score—0–15), moderate risk (Score—16–23), or low risk (score > 23). The inter-observer agreement regarding the scoring of each item for the included studies was determined using the kappa statistics [17]. Based on the power of kappa, the degree of agreement was defined as almost perfect (0.81–1.00), substantial (0.61–0.80), moderate (0.41–0.60), fair (0.21–0.40), or slight (0.00–0.20).

### 2.5. Statistical Analysis

The baseline data were represented as numbers, proportions, averages, and ranges. The meta-analysis was conducted using RevMan 5.4 (Cochrane Collaboration, London, UK). For the dichotomous outcomes, the risk ratios (RR) with 95% confidence intervals (CI) were estimated. The Mantel–Haenszel (M-H) method was applied for the calculation of pooled risk ratios. For the continuous outcomes, the mean difference (MD) with a 95% CI was estimated. Subsequently, the weighted mean difference (WMD) was calculated using the inverse variance (IV) method. The level of heterogeneity among the included studies was evaluated using the I^2^ statistics. A random-effects model was used in case of substantial heterogeneity (I^2^ >50%). A *p*-value of <0.05 was considered statistically significant.

## 3. Results

### 3.1. Study Characteristics

**Figure 1:**
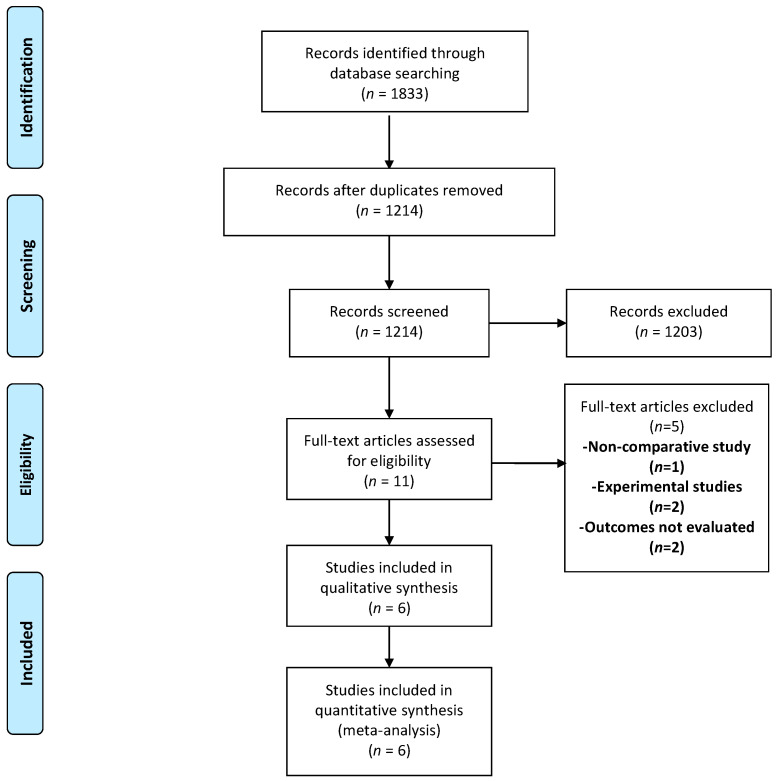
Selection of the relevant studies using the Preferred Reporting Items for Systematic Review and Meta-Analysis (PRISMA) flow diagram.

Out of 1833 records identified, 619 duplicate articles were removed. The remaining 1214 articles were screened for eligibility. Of these, 1203 abstracts were excluded and only eleven full texts were assessed for inclusion (Figure 1). One of them was a non-comparative study and was excluded [18]. Similarly, two other studies were excluded as they had used resected bowel specimens for comparison and no clinical outcomes were measured in these [19,20]. Additionally, two studies had not evaluated any of the outcomes and were thus also excluded [21,22]. Therefore, only six studies were included in the final meta-analysis [13,23,24,25,26,27]. The study designs of these studies were retrospective (*n* = 3), prospective (*n* = 2), and ambispective (*n* = 1). Three studies each demonstrated the outcomes after the division of the mesoappendix [25,26,27] and securing of the appendicular base, respectively [13,23,24]. The baseline characteristics of the included studies are presented in Table 1.

### 3.2. Summary of the Included Studies

**Aydogan et al., 2009** [23]

This was a retrospective comparative study from Turkey and compared the use of LigaSure and endoclips in laparoscopic appendectomy. The study population comprised patients undergoing laparoscopic appendectomy with histological proof of acute appendicitis in the operative specimen. The mean operative time was shown to be significantly shorter in the LigaSure group (41 ± 21 min V/s 54 ± 26 min, *p* < 0.05). Moreover, the conversion rate was also lesser in the LigaSure group (9.4% V/s 11.2%, *p <* 0.05). However, there was no significant difference in the hospital stay (1.5 days V/s 1.5 days) and the overall postoperative complication rate (2.3% V/s 2.6%).

**Sucullu et al., 2009** [25]

This was a randomized study from Turkey that compared the use of LigaSure with that of endoclips for securing the mesoappendix. They compared these two groups primarily in terms of operative duration and hospital stay. The results showed that the operative duration was significantly lower in the LigaSure group (49.06 ± 14.72 min V/s 59.69 ± 12.54 min, *p* = 0.036); however, no significant difference was noted in the mean hospital stay (2.5 ± 1.13 days V/s 2.6 ± 0.93 days). The additional analgesic need in both groups was, however, comparable.

**Lee et al., 2014** [26]

Conducted in South Korea, this was a retrospectively conducted study that compared clipless securing of mesoappendix (Harmonic/electrocautery) with that of endoclip. The overall operative time was significantly lower in the Harmonic scalpel group as compared to the electrocautery and endoclip groups (51.4 ± 25.6 min V/s 57.8 ± 25.7 min V/s 58.1 ± 24.9 min, *p* = 0.002). The duration of hospital stay (11.9 ± 4.5 days V/s 11.4 ± 4.5 days V/s 11.8 ± 6.8 days; *p* = 0.424) and the complication rate (1.3% V/s 1.2 % V/s 1.7%, *p* = 0.476) were, however, comparable in all three groups. They also compared the cost-effectiveness of all three methods with the effective cost for each being USD 571 for the endoclip group, USD 959 for the harmonic scalpel group, and USD 452 for the electrocautery group.

**Park et al., 2019** [27]

This study was a retrospective cohort study from South Korea and evaluated the postoperative outcomes in patients of acute appendicitis following ligation or division using either Harmonic scalpel or endoclips/Hem-o-Locks/laparoscopic staples. The operative time was again significantly lesser in the Harmonic group (37.6 ± 15.4 min V/s 48.6 ± 17.9 min, *p* < 0.001). The percentage of patients requiring indwelling catheters (8.6% V/s 10.4%, *p* = 0.398) was comparable in both groups. Additionally, the estimated blood loss (18.6 ± 13.54 mL V/s 20.7 ± 14.4 mL, *p* = 0.032) and rate of appendicular perforation (33.1% V/s 45.8%, *p* < 0.001) were both significantly lesser in the Harmonic group. The overall postoperative complication rate also had a nonsignificant difference between the two groups (6.9% V/s 5.4%, *p* = 0.356). However, on subdividing the type of complications, the rate of postoperative ileus (18.1% V/s 31.6%, *p* = 0.025) was significantly lower in the Harmonic group. The rates of other specific complications, i.e., wound seroma (2.1% V/s 7.7%, *p* = 0.784), wound infection (3.1% V/s 1.7%, *p* = 0.145), diarrhea (0.7% V/s 0.3%, *p* = 0.343), small bowel obstruction (0.3% V/s 0.7%, *p* = 0.516), and intra-abdominal abscess (1% V/s 1.2%, *p* = 0.780), were comparable between both the groups. The mean postoperative hospital stay was also comparable between the two groups (3.8 ± 1.3 days V/s 3.8 ± 1.2 days, *p* = 0.772).

**Gupta et al., 2020** [13]

This study was conducted in India and was ambispective in nature. The authors aimed to evaluate the outcomes of the sealing of the appendicular base with Harmonic vis-à-vis sealing with a ligature. The mean operative time was shown to be significantly shorter (28.46 ± 7.19 min V/s 43.34 ± 6.7 min, *p* < 0.001) in the Harmonic group as compared to the ligature group. The postoperative complication rate was found to be comparable in both groups.

**Pogorelić et al., 2021** [24]

This was a prospective bicentric study conducted in Croatia and Egypt and compared the clinical outcomes of patients undergoing securing of the appendicular base using either a Harmonic scalpel or polymeric clips. The study demonstrated that the Harmonic group had a lesser incidence of postoperative complications (0 V/s 5.1%) and fever (6% V/s 14.2%). The duration of surgery (21 min V/s 30 min, *p* < 0.0001) as well as the postoperative hospital stay (2 days V/s 3 days, *p* < 0.0001) were also significantly lesser in the harmonic group. However, on subdividing the hospital stay by complication status, it was noted that in patients with simple appendicitis, there was no significant difference (2 days V/s 2 days, *p* = 0.390) between the two groups.

### 3.3. Quality Assessment

A detailed methodological quality assessment is depicted in Table 2. The average scores assigned to the included studies ranged from 14.5 to 19.5. The studies by Gupta et al. and Park et al. had the maximum and minimum risk of bias, respectively [13,27]. While one study had a high risk of bias (Gupta et al.), the risk was moderate in the remaining five studies [23,24,25,26,27]. The inter-observer agreement for quality assessment was almost perfect (kappa = 0.8918, *p* < 0.0001).

### 3.4. Meta-Analysis

**(a)**  **Surgical site infections (SSI)/Intra-abdominal collections**

**SSI/Intra-abdominal collections with respect to appendicular base ligation**

**Figure 2:**
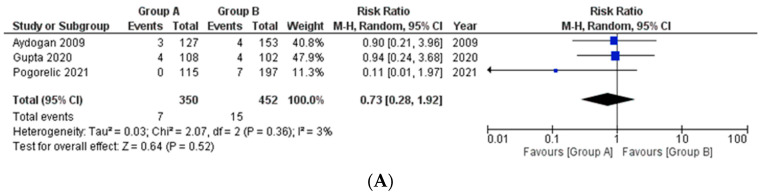

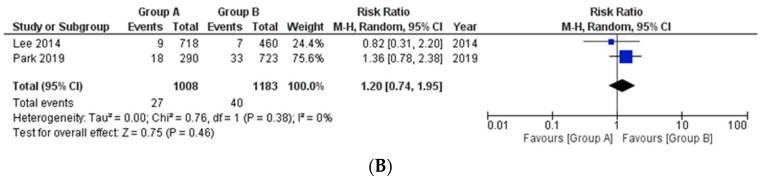
Forest plot comparison between the two patient groups (group A included cases wherein an energy device was used while group B included patients in whom suture or Endoclip or Hem-o-lok^®^ was used) in terms of the incidence of SSI/Intra-abdominal collections. Figure 2A, B depict the outcome comparison with respect to appendicular base ligation and mesoappendix division, respectively. Abbreviations: SSI, surgical site infections; M-H, Mantel–Haenszel method; CI, confidence interval.

This outcome was reported by three studies only (Figure 2A). A total of 350 and 452 patients were included in Group A and Group B, respectively. Of these, 7 patients had SSI/Intra-abdominal collections in Group A, whereas the same was reported in 15 patients in Group B. The pooled risk ratio showed no significant difference in the incidence of infections between the two groups (RR 0.73, 95% CI 0.28 to 1.92, *p* = 0.52). The estimated heterogeneity among the included studies was not substantial (I^2^ = 3%, *p* = 0.36).

**SSI/Intra-abdominal collections with respect to mesoappendix division**

The outcome was reported by only two studies (Figure 2B). A total of 1008 patients were included in Group A and 1183 patients were included Group B. Of these, 27 patients had SSI/Intra-abdominal collections in Group A, whereas the same was reported in 40 patients in Group B. The pooled risk ratio showed that there was no significant difference in the incidence of infections between both these groups either (RR 1.2, 95% CI 0.74 to 1.95, *p* = 0.46). The estimated heterogeneity among the included studies was neither substantial nor significant (I^2^ = 0%, *p* = 0.36).

**(b)**  **Postoperative Ileus**

**Ileus with respect to appendicular base ligation**

**Figure 3:**
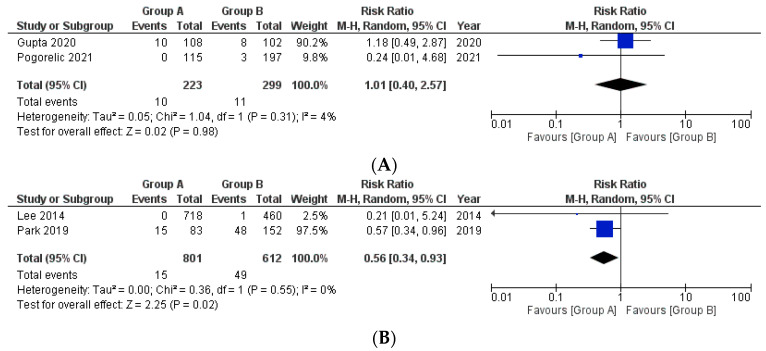
Forest plot comparison between the two patient groups (group A included cases where energy device was used while group B included patients in whom suture or Endoclip or Hem-o-lok^®^ was used) in terms of the occurrence of postoperative ileus. Figure 3A, B depict the outcome comparison with respect to appendicular base ligation and mesoappendix division respectively. Abbreviations: M-H, Mantel–Haenszel method; CI, confidence interval.

Two studies reported the given outcome (Figure 3A). The incidence was compared across 223 patients of Group A and 299 patients of Group B. Of these, 10 patients were reported to have postoperative Ileus in Group A, whereas the same was reported in 11 patients in Group B. Here also, the pooled risk ratio was not significant in the incidence for both the groups (RR 1.01, 95% CI 0.40 to 2.57, *p* = 0.98). The estimated heterogeneity among the included studies was also not substantial (I^2^ = 4%, *p* = 0.31).

**Ileus with respect to mesoappendix division**

This outcome was reported by two of the included studies (Figure 3B). A total of 801 and 612 patients were included in Group A and Group B, respectively. Of these, 15 patients had postoperative ileus in Group A, whereas 49 patients in Group B reported the same. The pooled risk ratio showed that a statistically significant difference was present in the postoperative ileus in patients belonging to Group A and Group B (RR 0.56, 95% CI 0.34 to 0.93, *p* = 0.02). The estimated heterogeneity among the included studies was not substantial (I^2^ = 0%, *p* = 0.55).

**(c)**  **Operative Time**

**Operative time with respect to appendicular base ligation**

**Figure 4:**
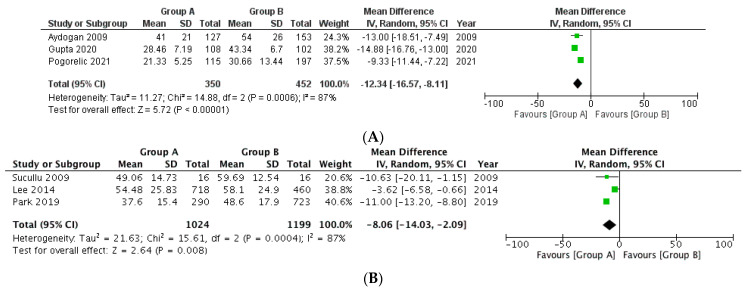
Forest plot comparison between the two patient groups (group A included cases where energy device was used while group B included patients in whom suture or Endoclip or Hem-o-lok^®^ was used) in terms of the average operative time (minutes). Figure 4A, B depict the outcome comparison with respect to appendicular base ligation and mesoappendix division, respectively. Abbreviations: SD, standard deviation; IV, inverse variance method; CI, confidence interval.

A total of three studies were included in this analysis with a total of 350 patients in Group A and 452 patients in Group B, respectively (Figure 4A). The mean difference between both the groups was significant with the operative time being lesser in Group A (MD −12.34, 95% CI −16.57 to −8.11, *p* < 0.00001). The heterogeneity of the included studies was, however, significantly substantial (I^2^ = 87%, *p* = 0.0006).

**Operative time with respect to mesoappendix division**

The three studies included a total of 1024 patients and 1199 patients in both Group A and Group B respectively (Figure 4B). The mean difference of the operative time between both Group A and Group B was again statistically significant (MD −8.06, 95% CI −14.03 to −2.09, *p* = 0.008); however, there was a presence of substantial heterogeneity among the included studies (I^2^ = 87%, *p* < 0.0004).

**(d)**  **Hospital stay**

**Hospital stay with respect to appendicular base ligation**

**Figure 5:**
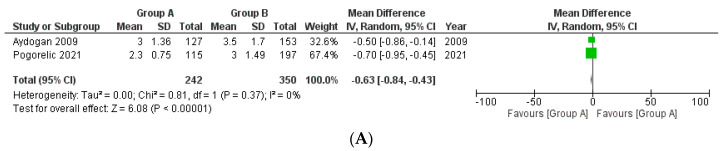

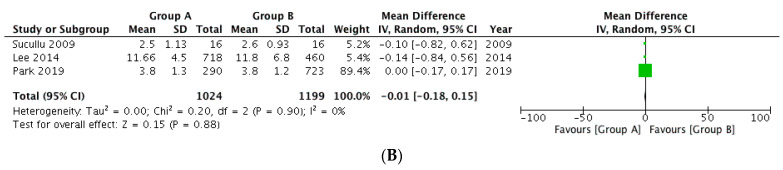
Forest plot comparison between the two patient groups (group A included cases wherein an energy device was used while group B included patients in whom suture or Endoclip or Hem-o-lok^®^ was used) in terms of the average hospital stay (days). Figure 5A, B depict the outcome comparison with respect to appendicular base ligation and mesoappendix division, respectively. Abbreviations: SD, standard deviation; IV, inverse variance method; CI, confidence interval.

Two studies were included in this analysis with a total of 242 patients in Group A and 350 patients in Group B (Figure 5A). The mean difference between both the groups was again statistically significant with the stay being lesser in Group A (MD −0.63, 95% CI −0.84 to −0.43, *p* < 0.00001). The heterogeneity of the included studies was not substantial (I^2^ = 0%, *p* = 0.37).

**Hospital stay with respect to mesoappendix division**

A total of three studies were included in this analysis with a total of 1024 patients and 1199 patients in Group A and Group B, respectively (Figure 5B). The mean difference in hospital stay between both groups was not significant (MD −0.01, 95% CI −0.18 to 0.15, *p* = 0.88). The heterogeneity of the included studies was not substantial (I^2^ = 0%, *p* = 0.90).

## 4. Discussion

We show that laparoscopic appendectomy is safe with all of the techniques analyzed here. Outcome measures of infectious complications regarding appendiceal stump closure and mesoappendix division are not related to the method used. Ileus was less common with sealing devices. The operative duration and length of hospital stay were shorter in patients belonging to group A. Modern sealing devices are thus safe and shorten the operative durations.

Appendicitis affects all age groups but is more prevalent between 10 and 20 years of age [28,29]. Appendectomy is among the most performed acute surgeries globally [30]. Currently, laparoscopic appendectomy is safe and relatively standardized, but the method of appendiceal stump closure and mesoappendix division remains variable [3,31,32,33]. Intra-abdominal abscess formation rates of 8% are currently similar between open and laparoscopic appendectomy in children and adults [30,34]. Nataraja et al. reported a lower incidence rate of 2.9% in laparoscopic appendectomy in children, which is similar to our results [35]. The incidence of postoperative ileus was 4% in the study population and was higher in group B with respect to mesoappendix division. This contrasts with the reported incidence of 0.4% and 1.78% in patients undergoing laparoscopic appendectomy, as reported by Nataraja et al. and Li et al., respectively, but similar to the incidence of 3.5% and lower than the 9.6%, as reported by Low et al. and Neogi et al., respectively [3,33,35,36].

Sealing devices have been proposed as safer for stump closure but present a higher economic burden for surgery. Costs currently seem to hinder the more comprehensive implementation of sealing devices for surgery in general [37]. Indeed, medical costs are increasing worldwide, with surgical expenses constantly growing [38]. Standardization of surgical equipment in laparoscopic appendectomy has proven safe, economical, and efficient [39]. A combination of modern devices may provide the highest surgical value regarding the operative times and lowest costs, depending on how hospital expenses are constructed [40].

The operative duration should be taken into consideration, especially for children, as the length of anesthetic exposure should be minimized [41]. The operative duration is also essential as shorter durations of surgery lead to more efficient operation room utilization and enable more operations to be performed within a given time frame. Further, in institutions wherein the operation room usage is charged by the minute, these durations are crucial from an economic aspect. The operative durations analyzed here were shorter in group A using sealing devices and shorter than that reported by Neogi et al. [3]. It is also imperative that the attempt to shorten the operative duration must never obscure safety concerns. The primary focus must always be on safety, which comes first and foremost. In our data, all appendix stump closure methods were equally safe regarding postoperative outcomes.

The duration of hospital stay was significantly shorter within group A. A variable length of stay was observed between the studies overall, with a spectrum consisting of Aydogan et al. reporting an LOS of 1.5 days and Lee et al. reporting up to 11 days, respectively [23,26]. The length of hospital stay was, however, generally similar to as previously reported [3,33]. Therefore, these observed differences in the duration of hospital stay may not be translated into daily practice as they were mainly of the order of hours.

### 4.1. Application to Clinical Practice

Our results show that the techniques for appendiceal stump closure and mesoappendix division are equally safe with modern methods. Clipless–sutureless laparoscopic appendectomy using energy devices can safely be adapted into clinical practice. The device selection may be left to surgeon preference as current evidence shows no method being superior to the others.

### 4.2. Strengths and Limitations

The present meta-analysis has a few limitations. First, there is variable reporting of the outcomes in the included studies. Second, most of these studies were observational studies and had a moderate risk of bias. Aydogan et al., Gupta et al., Pogorelic et al., Lee et al., and Park et al. did not randomize patients into groups, and the operative method was based on the surgeon’s preference [13,23,24,26,27]. Sucullu et al. used a quasi-randomization method, which may skew results with respect to operative times and hospital stay regarding mesoappendix division [25]. Third, none of the included studies presented data on hospital costs or the quality of life. Fourth, the data on postoperative bleeding or appendix stump rupture was also not reported. Perioperative bleeding causes a hematoma, which presents ideal growing media for bacterial contamination. As there were no significant differences in postoperative infections, nor was the incidence higher than previously reported, it may be assumed that perioperative bleeding is not a problem in this cohort. The reporting of complications was, however, inconsistent, hampering definite conclusions. Fifth, the included studies did not differentiate between uncomplicated vs. complicated appendicitis, which may present as completely different operative situations. The study of Pogorelic et al. showed a high rate of complicated appendicitis, and the perforation rates in all studies varied between <10 and 49%, reflecting variations in the patient populations. Finally, none of the studies presented data on the need for reoperations or readmissions.

Although a previously published systematic review on appendectomy in adults has shown that none of the methods are superior to others in terms of appendiceal stump closure and mesoappendix division, it is notable that none of the included studies in that review utilized a Harmonic scalpel [6]. The present review included studies in which a Harmonic scalpel or other sealing devices were utilized. However, we did not embark on the subgroup analysis as the material for this was limited. Nevertheless, the results from this study indicate that novel techniques are safe and could be readily adapted for appendectomy at all ages.

## 5. Conclusions

Laparoscopic appendectomy is safe, and the occurrence of complications is not affected by the method of stump closure or mesoappendix division. Postoperative ileus seems to be less common while using energy devices for mesoappendix division. The average operative duration is significantly shorter if energy devices are utilized during laparoscopic appendectomy. Before any definite conclusions are drawn, future studies addressing individual devices with surgeons of similar levels need to be conducted, especially focusing on different age groups separately.

## Figures and Tables

**Table 1:** Baseline characteristics of the included studies. * includes 372 and 246 patients in whom Harmonic scalpel and monopolar cautery were, respectively, utilized.

Author	Study Design	Sample Size (*n*)		Gangrenous/Perforated Appendicitis (*n*)		Converted to Open Approach (*n*)	
		A	B	A	B	A	B
Aydoganet al., 2009 [23]	Retro	127	153	30	38	12	17
Suculluet al., 2009 [25]	Pro	16	16	3	3	0	0
Lee et al., 2014 [26]	Retro	718 *	460	126	67	1	1
Park et al., 2019 [27]	Retro	290	723	21	35	0	1
Gupta et al., 2011 [13]	Ambi	108	102	**	**	-	-
Pogorelić et al., 2021 [24]	Pro	115	197	52	96	-	-

** Patients with perforated appendicitis were excluded. Abbreviations. Retro, retrospective study. Pro, prospective cohort. Ambi, ambispective study.

**Table 2:** Independent methodological quality assessment by two observers utilizing the Downs and Black scale.

Assessment by Observer 1						
Study	Reporting	External Validity	Internal Validity- Bias	Internal Validity-Confounding	Power	Total Scores
Aydogan et al., 2009 [23]	7	3	4	2	0	16
Sucullu et al., 2009 [25]	8	3	4	3	0	18
Lee et al., 2014 [26]	8	3	4	2	0	17
Park et al., 2019 [27]	8	3	5	3	0	19
Gupta et al., 2020 [13]	6	3	4	1	0	14
Pogorelić et al., 2021 [24]	8	3	5	3	0	19
**Assessment by observer 2**						
**Study**	**Reporting**	**External validity**	**Internal validity- bias**	**Internal validity-confounding**	**Power**	**Total scores**
Aydogan et al., 2009 [23]	8	3	4	3	0	18
Sucullu et al., 2009 [25]	7	3	5	3	0	18
Lee et al., 2014 [26]	8	3	3	3	0	17
Park et al., 2019 [27]	9	3	5	3	0	20
Gupta et al., 2020 [13]	7	3	4	1	0	15
Pogorelić et al., 2021 [24]	8	3	5	3	0	19
**Cumulative assessment**						
**Study**	**Rater 1**	**Rater 2**	**Mean**	**Kappa value**	***p***	
Aydogan et al., 2009 [23]	16	18	17	0.8918	<0.0001	
Sucullu et al., 2009 [25]	18	18	18			
Lee et al., 2014 [26]	17	17	17			
Park et al., 2019 [27]	19	20	19.5			
Gupta et al., 2020 [13]	14	15	14.5			
Pogorelić et al., 2021 [24]	19	19	19			

## Data Availability

Study data are available from authors on reasonable request.
